# Efficacy of electronic apex locators in comparison with intraoral radiographs in working length determination- a systematic review and meta-analysis

**DOI:** 10.1186/s12903-024-04259-w

**Published:** 2024-05-04

**Authors:** Gurveen Kaur, Anchu Rachel Thomas, Renu Sarah Samson, Eby Varghese, Ratna Rachel Ponraj, Sumanth Kumbargere Nagraj, Deepti Shrivastava, Hmoud Ali Algarni, Amna Yusuf Siddiqui, Osama S. Alothmani, Kumar Chandan Srivastava

**Affiliations:** 1 Dept of Conservative Dentistry and Endodontics, National Dental College, Baba Farid University of HEALth Sciences, Faridkot, India; 2 Department of Conservative Dentistry and Endodontics, Faculty of Dentistry, Manipal University College Malaysia, Jalan Batu Hampar, Bukit Baru, Melaka, Malaysia; 3 Department of Preventive Dentistry, College of Dentistry, Jouf University, 72345 Sakaka, Saudi Arabia; 4 Department of Restorative Dental Sciences, College of Dentistry, Jouf University, 72345 Sakaka, Saudi Arabia; 5 Department of Endodontics, Faculty of Dentistry, King Abdulaziz University, Jeddah, Saudi Arabia; 6 Oral Medicine & Radiology, Department of Oral & Maxillofacial Surgery & Diagnostic Sciences, College of Dentistry, Jouf University, 72345 Sakaka, Saudi Arabia; 7 Department of Oral Medicine & Radiology, Saveetha Dental College, Saveetha Institute of Medical and Technical Sciences, Saveetha University, Chennai, 602105 India

**Keywords:** Endodontic, Root canal treatment, Working lengt, Radiographs, Apex locator

## Abstract

**Background:**

Successful endodontic treatment needs accurate determination of working length (WL). Electronic apex locators (EALs) were presented as an alternative to radiographic methods; and since then, they have evolved and gained popularity in the determination of WL. However, there is insufficient evidence on the post-operative pain, adequacy, and accuracy of EALs in determining WL.

**Objective:**

The systematic review and meta-analysis aims to gather evidence regarding the effectiveness of EALs for WL determination when compared to different imaging techniques along with postoperative pain associated with WL determination, the number of radiographs taken during the procedure, the time taken, and the adverse effects.

**Methods:**

For the review, clinical studies with cross-over and parallel-arm randomized controlled trials (RCTs) were searched in seven electronic databases, followed by cross-referencing of the selected studies and related research synthesis. Risk of bias (RoB) assessment was carried out with Cochrane's RoB tool and a random-effects model was used. The meta-analysis was performed with the RevMan software 5.4.1.

**Results:**

Eleven eligible RCTs were incorporated into the review and eight RCTs into the meta-analysis, of which five had high RoB and the remaining six had unclear RoB. Following meta-analysis, no significant difference in postoperative pain was found among the EAL and radiograph groups (SMD 0.00, CI .29 to .28, 354 participants; *P* value = 0.98). Radiograph group showed better WL accuracy (SMD 0.55, CI .11 to .99, 254 participants; *P* value = 0.02), while the EAL group had 10% better WL adequacy (RR 1.10, CI 1.03–1.18, 573 participants; *P* value = 0.006).

**Conclusion:**

We found very low-certainty evidence to support the efficacy of different types of EAL compared to radiography for the outcomes tested. We were unable to reach any conclusions about the superiority of any type of EAL. Well-planned RCTs need to be conducted by standardizing the outcomes and outcome measurement methods.

**Supplementary Information:**

The online version contains supplementary material available at 10.1186/s12903-024-04259-w.

## Introduction

Successful endodontic treatment is highly dependent on efficient debridement, disinfection, and three-dimensional obturation [1, 2]. Additionally, precise working length (WL) determination is also considered as a crucial step [3, 4]. WL is the measurement from a reference at the coronal portion of the tooth to a specific location where the root canal procedures should conclude [5].

Root canal instrumentation and obturation are usually terminated at the WL, the best approximation to the apical constriction (AC), regarded as the anatomic reference [6, 7]. This prevents damage to the peri-radicular tissue [8, 9]. Incomplete obturation could leave infected tissue in the apical region and prevent healing of the periapical region [10–12]. However, it is challenging to identify the AC clinically or radiographically because it is a highly variable histological reference [13, 14].

Radiographic method is the most preferred method for locating the apical end of the roots. However, accurate interpretation is often challenging with two-dimensional radiographs due to the superimposition of anatomical structures [15]. Concerns regarding radiation exposure, the number of radiographs taken, and the time required to acquire radiographs [16–18].

Apex locators have been used as an effective alternative for determination of WL compared to the radiographic method. Initially, these devices evaluated electrical resistance and, later, relative impedance within the root canal. They are referred to as "Foramen locators" to clarify their function in the WL determination of the canal. Furthermore, due to their ability to measure relative impedance inside the root canal, these devices can also identify the alteration in the cross-sectional area of the canal near its exit, commonly referred to as the "apical constriction" [19].

Nevertheless, the reliability of measurements is often compromised by the presence of fluids and metallic restorations. Regardless of the drawbacks, EAL are increasingly used in clinical practice because they reduce the number of radiographs and treatment time. However, a consensus is needed on the comparative accuracy of electronic and radiographic methods, as there is insufficient evidence-based research.

This systematic review and meta-analysis examine the evidence for the efficacy of EALs in assessing postoperative pain, adequacy, and accuracy in determining WL compared with various imaging modalities in patients with permanent dentition. The number of radiographs taken during the procedure, time required, and associated adverse effects were also evaluated.

## Materials and methods

This systematic review and meta-analysis was performed and written based on the PRISMA guidelines for reporting research synthesis [20, 21]. Protocol was recorded in an international database (PROSPERO- ID: CRD42021254714).

### Eligibility criteria

The eligibility criteria were designed according to PICO (Patient/Population, Interventions, Comparison, Outcomes). The patient population included permanent human teeth with closed apices undergoing root canal treatment. The intervention included EALs or Endo motor with integrated apex locator; comparator included 2-dimensional intraoral periapical radiographs and 3-dimensional imaging (CBCT). The primary outcome was postoperative pain, WL accuracy, and adequacy, and secondary outcome included the number of radiographs, time required, and associated adverse effects. The study question was: Is there any difference in postoperative pain and working length determined using apex locators compared to other imaging modalities?

Included studies involved fully developed human permanent teeth; clinical studies; studies that provide comprehensive data about the measured distance between the file tip employed for EAL measurement and the exact location of the apical constriction (AC); manual assessment of the working length using multiple frequency EALs and determination of the working length during rotary file preparation.

Studies done on teeth with open apex, primary teeth, teeth with resorption, perforated, resected teeth; endodontically treated teeth; studies with an observing of the file through the apex; case reports, reviews, and observational studies; histological evaluation of apical anatomy; studies in which the distance of the file tip used for EAL measurement to the AC is not specified or given as a range, first and second generation EAL; identification of landmarks other than the constriction/ minor foramen; foreign language articles without English translation were excluded.

### Strategy for search and selection of studies

A computerized literature search in seven databases was undertaken: MEDLINE PubMed, MEDLINE via OVID, LILACS, Embase, Scopus, Google Scholar, Cochrane Library from 1990 until October 2023, using words ‘‘Radiography’’, ‘‘Working length’’, and ‘‘Electronic Apex Locator’’ through PubMed to find the Medical Subject Headings terms for each word (Table 1). Additionally, a cross-reference search was done for included studies, other systematic reviews, and meta-analysis through connectedpapers.com.

**Table 1:** Search strategy for the literature

Directory	MESH Terms	Filters & limits
MEDLINE via PubMed	((("apex locator") OR ("apex locators")) AND ("working length")) AND (((("cone beam computed tomography") OR (CBCT)) OR ("cone beam CT")) OR (((radiography) OR ("intraoral radiograph")) OR ("periapical radiograph")))	No filters
MEDLINE via OVID	#1. (apex adj5 locator*).mp #2. (working adj5 length).mp #3. Radiography/ or Humans/ or Adults/ #4. (periapical adj5 radiograph*).mp #5. (intraoral adj5 radiograph*).mp #6. Humans/ or exp cone beam computed tomography/ #7. 3 or 4 or 5 or 6 #8. 1 and 2 and 7	No filters
LILACS	Apex [Words] and locator[words]	No filters
Embase via OVID	#1. (apex adj5 locator”).mp #2. (working adj5 length).mp #3. radiograph*.mp #4. (periapical adj5 radiograph*).mp #5. (intraoral adj5 radiograph).mp #6. exp cone beam computed tomography/ #7.3 or 4 or 5 or 6 #8. 1 and 2 and 7	No filters
Scopus	TITLE-ABS (“apex locator”) AND (LIMIT-TO (DOC TYPE. “ar”) OR LIMIT TO (DOCTYPE. “cp”))	No filters
Google scholar	“Apex locator” AND “Radiograph” AND “Randomized controlled trial”	No filters
Cochrane Library	“apex locator” “working length”	No filters

Among the authors, two of them assessed the research headings and abstracts independently and in duplicate using Rayyan software [22]. For articles with full text, another two authors screened the studies individually and in duplicate to meet the inclusion and exclusion criteria. Differences in opinion were resolved through discussion with the arbiter to reach a consensus.

### Data collection process

Data extraction form was designed with information about the study, purpose, sample, intervention, comparator, outcome measurement, results, adverse events, and author's conclusion (Table 2). Excluded studies and grounds for omission are reported in Table 3.

**Table 2:** Data extraction of included studies in the systematic review

Study	Purpose	Sample	Intervention Group (Type Of eALs)	Comparator Group	Outcome Measurement	Results	Adverse Events	Author's Conclusion
Kara-Tuncer & Gerek (2014) [23]	Incidence of post-operative pain associated with eAL and radiographic method of WL determination	220 patients with single rooted teeth (114 patients—maxillary teeth and 106 patients—mandibular teeth)	ROOT ZX—Third generation	Digital radiograph	4-point pain severity rating: 1, absence of pain; 2, slight discomfort, no requirement for treatment; 3, pain alleviated with analgesics; and 4, pain and/or swelling not diminished by basic analgesics, necessitating urgent intervention	The post-surgical discomfort within the 4 to 48-h period under examination exhibited no statistically significant variance (*P* > 0.05) between the groups	Not reported	No difference in postoperative pain was observed between eAL and digital radiography groups
Naeem et al*.* (2017) [24]	Effect of WL determination using electronic apex locator (eAL) and digital radiography on postoperative pain and the quantity of analgesics consumed	54 patients (mandibular molars)	DENTA PORT ROOT ZX -Third generation	Digital radiograph	A questionnaire was given to record the intensity of the pain & the frequency of analgesic taken postoperatively at intervals of 4, 6, 12, 24 & 48 h	For postoperative pain the total pain score for eAL group was (0.96 ± 1.25) and digital radiography group was (0.73 ± 1.37), results were not statistically significant (0.29) (*P* > 0.05). For analgesic intake results were also statistically non-significant (*P* > 0.05) with values of (0.96 ± 1.24 & 0.73 ± 1.37) for both groups respectively	Not reported	No difference was observed in post-operative pain and amount of analgesics consumed between the eAL or digital radiography groups in multirooted teeth
Vanitha & Sherwood (2019) [25]	Assessment of clinical accuracy of readings at the apex and 0.5 marks of three different apex locators: IPEX II, ROOT ZX, and APEX ID, in comparison to intraoral radiographs	Sixty patients (mandibular first molar)	ROOT ZX—Third generation, APEX ID- Third generation, IPEX II-Fourth generation	Intraoral radiograph	APEX measurements (obtained via the eALs) were documented at 0.5 points, and these findings were subsequently verified using periapical X-rays	Prior to canal treatment, the distance between APEX and 0.5-mark for the three canals was 0.42, 0.62, and 0.43 mm, respectively, for IPEX II, ROOT ZX, and APEX ID The 0.5-mark of IPEX II and APEX ID were in closer proximity to the radiographically estimated working length compared to the readings from the ROOT ZX device	Not reported	Negligible differences were seen at the APEX measurements of the eALs in comparison with radiographic observations. When considering the 0.5-mark measurements, there was a significant disparity in WL estimation between the ROOT ZX and the IPEX II and APEX ID devices
Rathore et al*.* (2020) [26]	Evaluation of the accuracy of eAL in comparison with the tactile and conventional radiographic method for WL determination in primary and permanent teeth	30 children (permanent molars)	ROOT ZX—Third generation	Conventional Radiographs	For eAL, WL was measured using 0.5-mm precision endodontic ruler and for conventional radiograph, Grossman’s method of WL determination was used	Mean WL readings for MB, ML, DB and DL for the Eal were 19.41 ± 0.87, 19.02 ± 0.65, 19.9 ± 0.75, and 19.52 ± 0.85, respectively and19.3 ± 0.83, 19.15 ± 0.69, 19.83 ± 0.86, and 19.34 ± 0.66, respectively for conventional radiograph group. The *p* values for MB and ML canals 0.04, 0.002 (statistically significant), and for DB and DL canals 0.84 and 0.48 (statistically insignificant)	Not reported	Determination of WL employing the eAL, radiographic technique, and tactile approach yielded analogous outcomes, with no statistically notable distinctions among the groups, except for the mesiolingual and distobuccal canals of the teeth
Singh et al*.* (2015) [27]	Effect of WL determination by using eAL or radiographic method on the adequacy of final WL	153 single rooted teeth	Raypex5—Fourth generation	Periapical radiograph	Evaluation of master cone radiographs were done and graded as (1) short (2 mm lesser than the radiographic apex), (2) acceptable (0–2 mm from the radiographic apex), and (c) over (beyond the radiographic apex)	A higher percentage of acceptable cases (92.1%) were observed with eAL as compared to radiographs (83.11%) Group 1, but the difference was not statistically significant	Not reported	Significantly less over cases with Raypex5 apex locator. While in acceptable and short cases, Raypex5 comparable to radiographic length measurement
Jarad et al. (2011) [28]	Efficacy of apex locators WL determination when compared to traditional WL radiographs	51 patients	Raypex 5 Fourth generation	Periapical radiograph	The primary outcome was the acceptability of the master gutta-percha cone (positioned within or no more than 2 mm from the radiographic apex).Other outcomes were the distance between the master cone GP and the root apex, and the total time taken for the procedure	Mean length of the master cone GP to the radiographic apex—1.06 mm (SD = 0.67) for the apex locator group, compared to 1.23 mm (SD = 0.72) in the periapical radiograph group (mean difference -0.18 mm, 95% CI -0.60 to 0.25)	Not reported	No significant difference was observed in WL determination using eAL and radiographs
Ravanshad et al*.* (2010) [29]	The effect of working length determination through electronic apex locator (eAL) or radiographic methods on the adequacy of the ultimate working length and its impact on the final obturation	84 patients with 188 canals	Raypex5-Fourth generation	Periapical radiograph	The adequacy of the master cone length and the final obturation length were categorized into three levels: 1. Short (less than 2 mm from the radiographic apex), 2. Acceptable (within 0–2 mm from the radiographic apex), and 3. Over (beyond the apex)	Acceptable cases for master GP Radiograph group = 82.1% and eAL group = 90.4% whereas for final obturation radiography: Radiograph group = 85.7% and eAL group = 90.4%). The mean quantity of X-rays for the eAL group was 3, whereas it was 4.07 for the radiograph group (a difference of high statistical significance)	Not reported	For acceptable and short cases, the results for EAL group were comparable to radiographic group. Also, eAL can reduce radiographic exposure and overestimation of root canal length
Saraf et al. (2017) [30]	Evaluating the effectiveness of six distinct eALs in multirooted teeth using intraoral periapical radiographs	90 teeth with 270 canals	ROOT ZX II -Third generation; ROOT ZX mini- Third generation; RAYPEX-6 -Sixth generation; I-ROOT- Fifth generation; Romiapex-A15—Third generation; Sybron Endo mini apex locator-Third generation	Intraoral Periapical radiographs	The assessment of the file tip reaching the radiographic apex on the IOPA was categorized as follows: Acceptable = 0—1mm short, Short = > 1mm short, Long = Beyond the apex	Out of 270 canals, 233 (86.3%) of the canals presented with acceptable WL, 28 (10.4%) canals presented with short WL, and 9 (3.3%) canals exhibited long WL beyond the apex	Not reported	Combining radiograph and apex locators provided accurate working length and successful endodontics
Koçak et al. (2013) [31]	Evaluation of the clinical accuracy of traditional radiographic method, eAL and apex locating endodontic motor	120 patients with 283 root canals	Root ZX mini eAL—Third generation, Multifunctional Endodontic motor with integrated apex locator (VDW Gold)	Conventional Radiograph	The master cone (final) radiographs were graded as- Short (shorter than 2 mm from radiographic apex), acceptable (a range of 0–2 mm from the radiographic apex), and over (extending past the apex)	Adequate filling cases were recorded as 77 (81,9%), 80 (87,0%) and 81 (83,5%) for traditional radiographic method, eAL and apex locating endodontic motor respectively	Not reported	There was no statistical distinction identified among the three assessed techniques
Joseph (2019) [32]	Evaluating the clinical success of eAL and Radiographic method of working length determination	83 teeth with 208 canals	ROOT ZX Mini –Third generation	Digital radiograph	Primary outcome was acceptability of master cone GP and postoperative radiograph after obturation was the secondary outcome. Radiographic healing after 3months was the tertiary outcome	Significant differences were observed for frequency of overextension and accurate fit between the 2 groups	Not reported	No statistical disparity in the long-term success rate
Khan et al*.* (2021) [33]	Comparing mean time of postoperative pain dissipation between eAL and digital radiographic method of WL determination	80 patients with single-rooted teeth	ROOT ZX -Third generation	Digital radiograph	A questionnaire designed to document the intensity of pain and the extent of analgesic consumption 48 h following the procedure. Tactile Analog Scale was used for post-operative pain	The mean VAS score was 4.35 ± 0.39 and 4.27 ± 0.48 for the radiographic and EAL group respectively. also, mean time (hours) for pain dissipation was 25.83 ± 11.05 and 24.25 ± 7.40, respectively (not statistically significant (*p*-value 0.138)	Not reported	No statistical disparity was seen among the techniques

**Table 3:** Characteristics of excluded studies

Study ID	Reasons of exclusion
Paludo et al*.* (2012) [15]	The outcome does not represent the objectives of this research synthesis
ElAyouti et al*.* (2009) [16]	The outcome does not represent the objectives of this research synthesis
Keller et al*.* (1991) [17]	In-vitro study
Orosco et al*.* (2012) [18]	Methodology not meeting the review criteria
Thomas et al*.* (2003) [19]	In-vitro study
Hembrough et al*.* (1993) [20]	Second generation eAL
Fouad et al*.* (1993) [21]	Methodology not meeting the review criteria
Tarallo et al*.* (2018) [22]	Methodology not meeting the review criteria
Diniz-de-Figueiredo et al*.*(2020) [34]	The outcome does not represent the primary or secondary objective of this review
Himel & Cain (1993) [35]	Second generation eAL

### Risk of bias evaluation

RoB evaluation was carried out by authors in pairs of two employing the RoB 1 tool [34]. Analysis and data entry was done using Review Manager 5.4.1 software.

### Effect measures

Postoperative pain was expressed in the VAS scale as continuous data. If the scales used for postoperative pain were similar, results were described as mean differences (MD) with 95% confidence intervals (CI). If the scales were different, standardized mean difference (SMD) with 95% CI was used to pool data for similar outcomes from different trials. For the data that is continuous, the results were laid out as MD with 95% CI or SMD with 95% CI to incorporate data. The results for the data that is dichotomous, were laid out as risk ratios (RR) with 95% CI. Apart from reporting adverse effects, the rest of the secondary results were represented in the form of MD with 95% CI. Adverse effects were expressed qualitatively.

### Synthesis methods

The meta-analysis was carried out with RevMan 5.4.1 which used SMD, random effect model, and inverse difference method. For WL determination mean difference, random effect model, statistical heterogeneity using the visual method, I^2^ and Chi-square, and inverse difference method were used. All the results were presented for difference comparison. The I^2^ statistic was used to evaluate the percent variation among studies due to heterogeneity.

When faced with uncertainty or incomplete information, the researchers of the studies were reached out to via email. If there was no communication from the authors after 15 days, it was reported as an unclear RoB. We computed the absent information using alternative available data sources, including standard deviations (SDs), *P*-values, visual representations, and, when necessary, data from other studies. Subsequently, the data was re-analysed in accordance with the intention-to-treat (ITT) principle whenever possible. Nevertheless, none of the studies furnished adequate details to conduct the ITT analysis.

### Certainty of evidence

The certainty of evidence for each outcome was assessed using the GRADE framework through GRADEpro GDT [21].

## Results

### Study selection

**Fig. 1:**
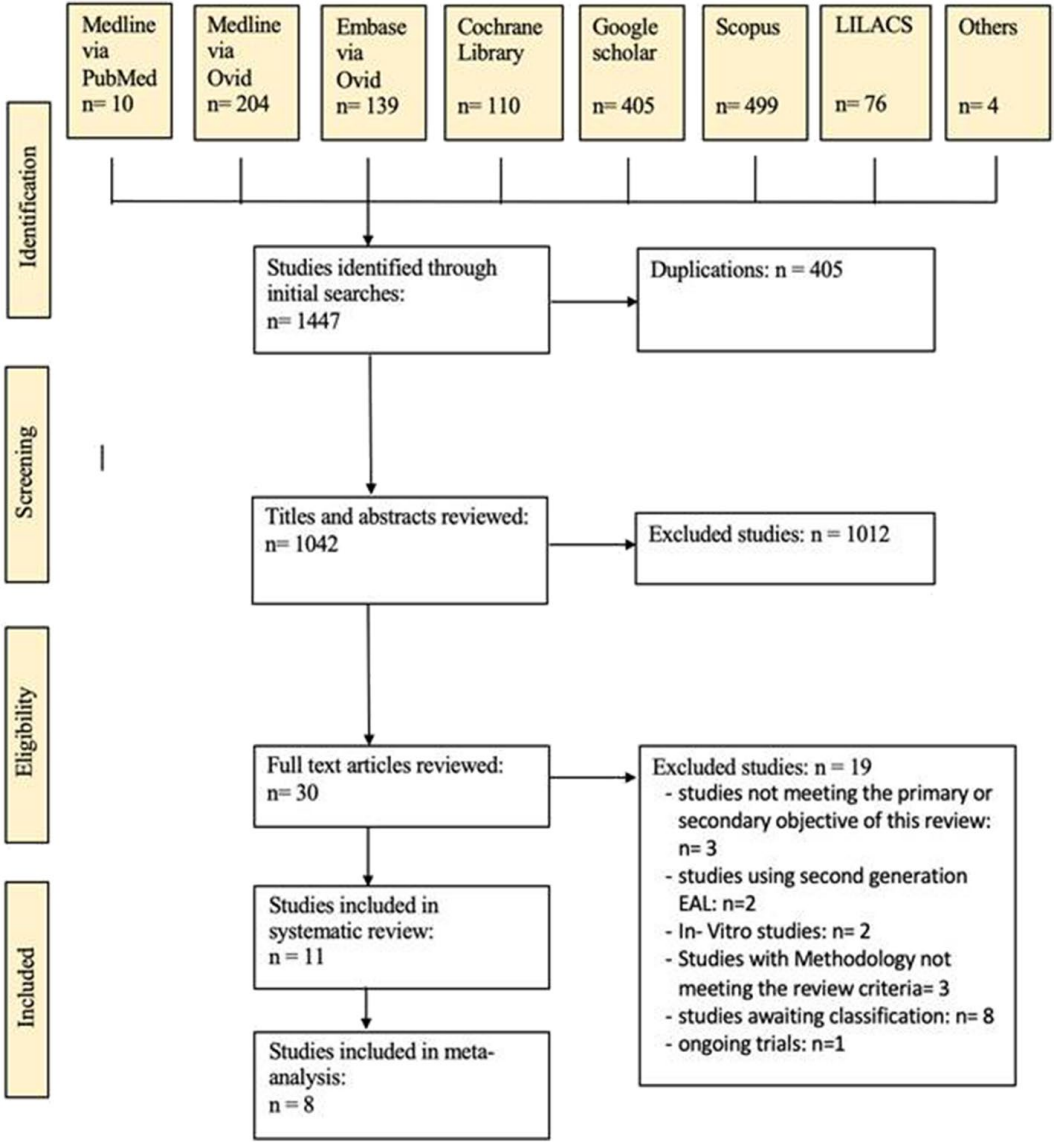
Flow diagram showing the literature selection process

One thousand four hundred forty-three references were found in the electronic database search and 4 additional studies from a non-systematic search of Google Scholar, contacting corresponding authors to attain full texts, relevant systematic reviews, and cross-referencing of included studies. Following the removal of duplicate entries, 1042 references underwent initial screening based on their titles and abstracts, resulting in the exclusion of 1012 references. From 30 full-text articles, 10 studies were excluded (Table 3) [19, 35–43]. One ongoing trial [44]was identified however, from 8 studies required data couldn’t be obtained and hence await classification [45–52]. In accordance with the specified inclusion criteria, 11 studies met the requirements for inclusion in the systematic review [23–33], with 8 of these studies being further incorporated into the meta-analysis [23, 24, 26–29, 31–33]. Figure 1 shows the study selection process in detail.

### Study characteristics

Among the eleven studies considered, ten studies were recorded in scholarly, peer-reviewed publications, while one among them was a dissertation [32]. Only two studies reported their funding details [28, 32]. The rest of the studies neither reported nor disclosed any funding details. All trials were parallel-group randomized controlled trials, with no cross-over trials.

In terms of patients, the least of the sample size was thirty [26], and the largest sample size was 220 (34). The maximum age of the participants taken was 75 years [32], and the lowest was 5 years [26]. All the selected studies had patients with permanent teeth except for one study, which had both primary and permanent dentition [26]. However, the results of the permanent teeth were only taken for analysis. One study [30] failed to provide any information about the age group.

Three studies [23, 27, 33] only included teeth with single roots and patent canals; however, another research [28] included both single-rooted and multirooted teeth. In addition, two research on multirooted teeth alone [24, 30] and two investigations on molars [25, 26] were also done. Root canals were considered in two studies; however, it was not stated whether the teeth were single or multirooted or whether they were mandibular or maxillary [29, 31].

Most included studies recruited healthy participants who were advised for root canal treatment. Patients with cardiac pacemakers, periapical radiolucency, curved roots, incomplete root formation, root resorption, Expectant mothers, and individuals with pre-existing systemic ailments were not considered for inclusion. One study did not mention any exclusion criteria [24].

### Risk of bias in studies

**Fig. 2:**
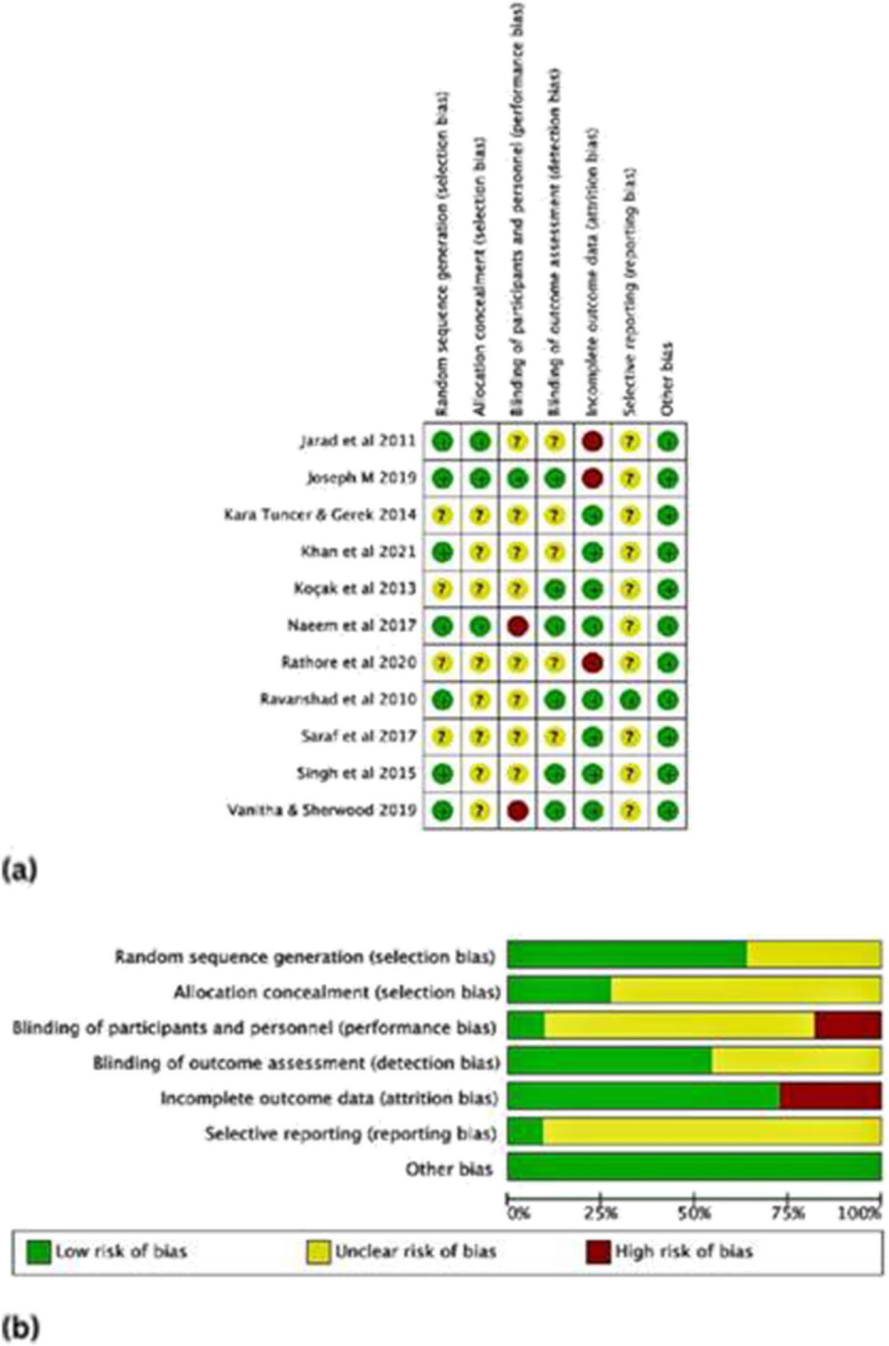
**a** Risk-of-bias summary. Review authors' judgements about each risk of bias item for each included study. Green colour indicates ‘low risk of bias’, yellow indicates ‘unclear risk of bias’ and red colour indicates ‘high risk of biases. **b** Review authors' judgements about each risk of bias item presented as percentages across all included studies. Green indicates ‘low risk of bias’, yellow indicates ‘unclear risk of bias’ and red indicates ‘high risk of biases

Five studies [24–26, 28, 32] had high susceptibility to bias and the rest of the 6 trials had unclear susceptibility to bias because they each had at least two unclear bias domains [23, 27, 29–31, 33] (Fig. 2a and b). Random sequence generation was reported in 7 trials [24, 25, 27–29, 32, 33] whereas, only 3 trials provided allocation concealment details [24, 28, 32]. One study provided details on the concealment of information from both participants and personnel [32] and blinding was reported in 6 studies [24, 25, 27, 29, 31, 32]. For the attrition bias, 3 trials [26, 28, 32] had drop-outs and hence had high susceptibility to bias. Among the entire set of studies, only one had a registered study plan and disclosed all the pre-planned results [29].

No other pertinent prejudices were identified in any of the 11 studies, rendering them at minimal risk of bias. All the incorporated studies had either higher risk or unclear risk of bias. Therefore, sensitivity analysis was not done as planned. Publication bias was not assessed since the meta-analysis didn’t include 10 or more studies.

### Results of individual studies

#### Meta-analysis

##### Postoperative pain

**Fig. 3:**
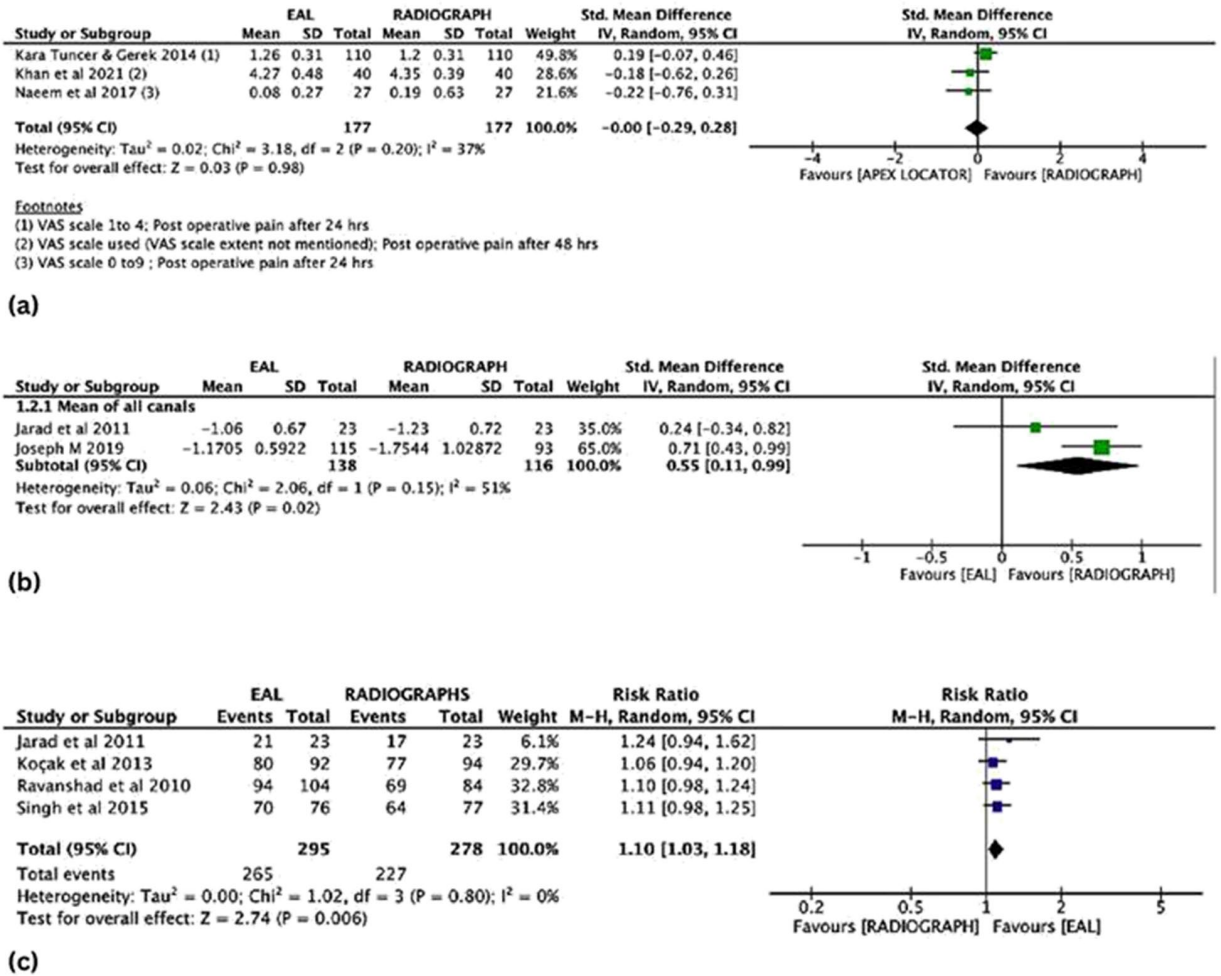
**a** Forest plot showing post-operative pain (Electronic Apex Locator Vs Radiographs). **b** Forest plot showing adequacy in WL determination (Dichotomous data). **c** Forest plot showing the accuracy of WL determination (Continuous data)

Three RCTs tested the postoperative pain following working length determination [23, 24, 33]. The inter-quartile range was derived from the graph using Plot Digitizer software for one study [23] and SMD was calculated according to Sect. 6.5.2.5 of the Cochrane Handbook [34]. The evidence showed no difference in postoperative pain in the EAL group in comparison with the radiograph group with a pooled effect estimate of SMD 0.00 (CI -0.29, 0.28, 354 participants; *P* value = 0.98). The confidence intervals intersected the effect line, making the findings' conclusion less robust (Fig. 3a).

##### Working length adequacy – dichotomous data

 Four studies evaluated the adequacy of working length determination between EAL and radiographs [27–29, 31]. The evidence showed better adequacy in working length determination in the EAL group in comparison to the group using radiographs with a pooled effect estimate of RR 1.10 (CI 1.03 to 1.18, 573 participants; *P* value = 0.02) (Fig. 3b). EAL also gives a 10% increase in working length adequacy compared to the radiographs.

##### Working length accuracy -continuous data

Four studies evaluated the WL accuracy [25, 26, 28, 32]. Two studies [25, 26] assessed the working length accuracy in individual canals and were not a part of the meta-analysis. The other two studies [28, 32] evaluated this comparison. The evidence suggests that working length determination in the EAL group in comparison to the group using radiographs with a pooled effect estimate of SMD 0.55 (CI 0.11 to 0.99, 254 participants; *P* value = 0.006) (Fig. 3c).

##### Certainty of evidence

The certainty of evidence was exceedingly minimal in accordance with the GRADE levels [21] of certainty for all the primary outcomes (Table 4). The downgrading was attributed to concerns about bias, inconsistency, and imprecision (as mentioned in Table 4).

**Table 4:** Summary of findings: eALs compared to radiographs in WL determination

Apex Locator compared to Radiographs in determining working length						
**Patient or population:** determining working length **Setting:** university hospitals **Intervention:** Apex Locator **Comparison:** Radiographs						
Outcomes	**Anticipated absolute effects** ^*^(95% CI)		Relative effect (95% CI)	Participants (randomized controlled trials)	Certainty level (GRADE)	Comments
	**Risk (Radiograph)**	**Risk (eAL)**				
Post-operative pain assessed with: VAS Scale from: 0 to 9 follow-up: range 24 h to 48 h	The range of post-operative pain is 0.19 to 4.35	SMD **0 SD** (.29 less to .28 higher)	-	354 (3)	⨁◯◯◯Very low^a,b,c^	
Accuracy in WL determination—Mean of all canals	The mean accuracy in WL determination—Mean of all canals was **-1.4922** mm	MD **0.55 mm higher** (0.11 higher to 0.99 higher)	-	254 (2)	⨁◯◯◯Very low^a,b,c^	
Adequacy in WL determination—Dichotomous	817 per 1,000	**898 per 1,000** (841 to 964)	**RR 1.10** (1.03 to 1.18)	573 (4)	⨁◯◯◯Very low^a,c^	
Adverse events	Not reported					

^*^The risk in the intervention group (and its 95% confidence interval) is according to the risk in the comparison group and the relative effect of the intervention (and its 95% CI).

*CI* confidence interval, *MD* mean difference, *RR* risk ratio, *SMD* standardised mean difference

GRADE Working Group grades of evidenceHigh certainty: we are very confident that the true effect lies close to that of the estimate of the effect

Moderate certainty: we are moderately confident in the effect estimate: the true effect is likely to be close to the estimate of the effect, but there is a possibility that it is substantially different

Low certainty: our confidence in the effect estimate is limited: the true effect may be substantially different from the estimate of the effect

Very low certainty: we have very little confidence in the effect estimate: the true effect is likely to be substantially different from the estimate of effect

Explanations

^a^Downgraded the level of certainty by two levels due to risk of bias

^b^Downgraded the level of certainty by two levels due to inconsistency

^c^Downgraded the level of certainty by one level due to imprecision

## Discussion

Adequate control of the working length during endodontic treatment is expected to impact the treatment results and prevent postoperative pain [53–55]. Electronic apex locators provide an effective means of locating working lengths for endodontic procedures [56]. The rationale of this review and meta-synthesis was to determine the existing body of evidence exhibiting the accuracy and adequacy of electronic apex locators and the postoperative pain during WL determination in comparison with radiographic methods which is prevalent in clinical use.

The recommendations of The European Society of Endodontology [57], suggest the use of an EAL followed by verifying the canal length with a radiograph during the procedure. In some cases, master cone radiograph to confirm the working length is suggested. Since none of the approaches can be considered an exact substitute for the histological method, the radiographic method has been used as the reference standard in this review. Histological methods cannot be a practical option when clinical trials are included.

The efficiency of EALs has been assessed in terms of postoperative pain, accuracy, and adequacy. Accuracy refers to the extent to which measurements deviate from a designated target, such as the apical foramen [58]. In this review, two studies [25, 26] mention the term accuracy in their clinical trials, although accuracy can only be compared using histological landmarks.

Master cone adequacy refers to when the master cone gutta-percha is considered adequate when it is 0–2 mm from the radiographic apex. Ng et al. studied the factors influencing the outcomes of endodontic therapy and concluded that every unistrumented millimeter of the canal, reduces the success rates by 12%, whereas overextended root fillings result in a 62% reduction in success [59]. Furthermore, in the study done by Meirinhos et al., they stated that periapical lesions were 3.1% more likely to be associated with short root fillings [60]. These points state the importance of the adequacy of the master cone. Hence, master cone adequacy should be an important outcome in the success of the therapy.

Based on the Cochrane Handbook, outcome measures are not considered criteria for including studies in a review [61]. Hence, the inclusion criteria for the research synthesis were designed based on the components: population, intervention, and comparator. Postoperative pain is a clinical outcome of inaccurate working length determination and master cone inadequacy; hence it was not included in the eligibility criteria.

A literature search involving seven electronic search engines and a cross-reference search was conducted to identify eligible research comparing the efficiency of EAL to radiographic methods to determine WL during root canal treatment of permanent teeth. Stringent criteria for inclusion and exclusion were employed in the studies to overcome the heterogeneity of data. The search terms were limited to “Radiography’’, ‘‘Working length’’, and ‘‘Electronic Apex Locator’’ to allow the inclusion of a larger number of studies. This systematic review included eleven in vivo studies with cross-over and parallel-arm randomized controlled trials from the above-mentioned period to ensure high-quality evidence. Eight of eleven studies were incorporated into the meta-analysis.

Intervention group incorporated, EALs representing third-generation and higher, and Endo motors with integrated apex locators due to their superior performance in comparison to the first and second-generation models that had been previously reported [28, 62, 63].

### Consensus and disparity with other systematic reviews:

The systematic review conducted by Amin et al*.* concluded that the accuracy of CBCT compared to EAL couldn’t be determined due to significant heterogeneity but suggested using pre-existing CBCT scans for WL determination [64]. A study concluded that the precision of EAL was comparable to the radiographic method. However, EAL and digital radiographic methods could reduce radiation dose exposure [24]. Another systematic review by Martins et al*.* reported inadequate scientific evidence and a considerable risk of bias. They suggested that WL determination using EAL could perform better than radiography alone, reducing patient radiation exposure. However, it was also recommended to perform at least one radiographic assessment to identify potential errors in electronic devices [56].

In this review, three out of eleven studies assessed the postoperative pain following WL determination using either EAL or radiographs [33, 62] which was the primary outcome. Kara-Tuncer & Gerek, Naeem et al*.,* and Khan et al*.* 2021 concluded no significant disparity among the EAL and digital radiography groups in the postoperative pain dissipation period [23, 24, 33].

The adequacy and accuracy of WL determination were interpreted as continuous and dichotomous data [25–29, 31–33]. Studies done by Rathore et al. and Vanitha & Sherwood were a part of the systematic review but weren’t incorporated in the meta-analysis as the readings presented were for individual canals [25, 26]. However, both the studies were included qualitatively and the RoB assessment was done. Substantial heterogeneity was observed (Heterogeneity: Tau^2^ = 0.06; Chi^2^ = 2.06, df = 1 (*P* = 0.15); I^2^ = 51%). For the dichotomous data, five studies evaluated the adequacy of working length between EAL and Radiographs [27–31]. However, the study done by Saraf et al. presented data with respect to six different EALs and thus not incorporated in the meta-analysis [30].

Appropriate utilization of EAL alone could eliminate the requirement for an additional radiograph for diagnosis to determine WL. Patients who don't need to repeatedly be exposed to radiation due to mental, medical, or dental issues may benefit from this procedure. According to previous research, the use of EALs decreased the frequency of taking radiographs, reducing treatment time, effort, and radiation exposure to patients [29, 65, 66]. Furthermore, EALs have the potential to reduce the incidence of overextension of root canal procedures, which may result in postoperative pain and difficulty in maintaining the apical stop. Estimation of WL prior to radiographic verification maintains the correct working length for termination of obturation thus preventing overestimation of root canal length resulting in postoperative pain. The results of a study suggested that the apical foramen was accurately located by apex locators when used correctly and that only one preoperative radiograph was required [67]. They also help the operator in suspecting root fractures, resorptions, and perforations [58].

There is significant evidence that shows the point of termination of root canal instrumentation and obturation affects the outcome of endodontic treatment. However, there is insufficient data on the outcome of endodontic therapy in relation to EAL assessment. As a result, the radiographic WL measurement remains crucial for clinical purposes, and the Ideal application of EALs would be to reduce the number of radiographic exposures by estimating the WL accurately before taking any radiographic measurements [68]. By considering both radiographic and electronic measurements, as well as the accuracy of EALs and the morphology of the root apex, the final working length (FWL), would be determined [65].

### Grade assessment and summary of findings

The meta-analysis suggested that radiographs were more accurate and EALs were more adequate in determining WL. Nonetheless, deriving dependable conclusions wasn’t feasible given the extremely low certainty of evidence owing to concerns about bias, inconsistencies, and imprecision. As a result, the superiority of any intervention over another couldn’t be determined. The findings of the research should be interpreted with caution and further clinical trials are needed to confirm the results.

Since the available evidence is of very low certainty, more randomised controlled trials assessing the effect of working length on postoperative pain, working length accuracy and adequacy need to be conducted. Also, more trials on the effect of integrated apex locators on the working length accuracy and adequacy needs to be performed. Furthermore, well- executed RCTs need to be carried out on different generations of EALs and radiographic methods such as digital radiography and CBCT. Moreover, the studies included in the review did not assess the cost-effectiveness, reduction in radiation exposure, and the number of radiographs except one study [29].

Due to the unavailability of the full text, eight articles weren’t incorporated in the research synthesis. The studies were searched across libraries, Research Gate, and Google Scholar, apart from efforts made to contact authors by email. The extensiveness of the search was limited since the grey literature was not explored. None of the included studies fulfilled the secondary outcome of adverse events.

To explore the possible effect of losses to follow-up on the effect estimates for the primary outcomes, sensitivity analyses, subgroup analysis and other factors for heterogeneity was planned in the protocol. Nevertheless, only one or two studies were included under most of the comparisons and thus the sensitivity and subgroup analysis could not be conducted.

The publication bias could not be assessed since the meta-analysis didn’t include 10 or more studies. The primary outcome, postoperative pain could have several contributory reasons. However, this review does not include all the reasons since the primary studies did not report the cause of the pain. The results of unpublished data have been included in this meta-analysis, since the overall evidence is of very low quality; we assume it wouldn’t have affected the results.

Future research should focus on:

- Population – Well-defined inclusion criteria for participants and clinical trials involving both anterior and posterior teeth with vital and necrotic pulps.
- Intervention –Trials focussing on evaluating intervention methods like Endo-Motors with integrated apex locators and the latest generation of EALs.
- Control- additional RCTs carried out on different generations of EALs and radiographic methods such as digital radiography and CBCT.
- Outcome- Trials focussing on assessing patient-related outcomes, adverse events, and direct outcome measurements like post-operative pain following WL determination.

The current review emphasizes the requirement for well-executed RCTs with trial reports adhering to the guidelines from CONSORT 2010 [69] and incorporating the results generated through fundamental consortiums like Core Outcome Measures in Effectiveness Trials (COMET) [70]. This will contribute to the current body of evidence, allowing researchers to formulate more dependable findings.

## Conclusion

The review concluded that there was no significant disparity with regard to post-operative pain in the EAL group compared to the radiograph group. Better accuracy with respect to WL using radiographs than EAL and better adequacy in WL using EAL than radiographs. Although there is no gold standard, adequacy is an important outcome in root canal treatment. Hence, we will be valuing the clinician’s judgment even for a short range of 0-2mm for acceptability.

### Supplementary Information

**Supplementary Materials 1.**

## Data Availability

The data will be available on reasonable request from the corresponding author.
